# An Improved Method for *Agrobacterium*-Mediated Genetic Transformation of Three Types of Lettuce

**DOI:** 10.3390/plants14040620

**Published:** 2025-02-18

**Authors:** Meghan C. Roche, Wusheng Liu, Ricardo Hernández

**Affiliations:** Department of Horticultural Science, North Carolina State University, Raleigh, NC 27607, USA; mcroche@ncsu.edu

**Keywords:** *Agrobacterium tumefaciens*, transformation, regeneration, *Lactuca sativa* L.

## Abstract

Lettuce genetic transformation is genotype-dependent. In the present study, we have successfully developed an optimized *Agrobacterium*-mediated transformation protocol for elite lettuce cultivars, which belong to the romaine, leaf, and butterhead cultivar types. We optimized the type and concentration of plant growth regulators (PGRs) and selection antibiotics and found that the use of 1-naphthaleneacetic acid (NAA; 0.10 mg/L) and 6-benzyladenine (BA; 0.25 mg/L) as plant growth regulators, the use of hygromycin (15 mg/L) for transgenic plant selection, and the use of cotyledons and the first true leaf as explants efficiently yielded transformed plants for seven out of the eleven tested cultivars, achieving a 24.3–100% transformation efficiency. These seven cultivars include two romaine-type cultivars, three leaf-type cultivars, and two butterhead-type cultivars, and mark the first successful genetic transformation of the romaine cultivars ‘Kahu’ and ‘Rosalita’, the leaf cultivars ‘Red Sails’ and ‘Royal Oak Leaf’, and the butterhead cultivar ‘Lollo Biondo’. We also observed that substituting hygromycin selection with kanamycin selection (40 mg/L) resulted in a 64.3% transformation efficiency in the butterhead-type ‘Mariska’, one of the remaining four cultivars. Our newly optimized protocols are applicable in elite lettuce cultivars for *Agrobacterium*-mediated genetic transformation and regeneration, enabling hygromycin or kanamycin selection.

## 1. Introduction

Lettuce (*Lactuca sativa* L.) is a diploid (2*n* = 2x = 18) species within the Asteraceae family [1] and a popular vegetable for production and consumption worldwide. Lettuce cultivation does not come without challenges, however, as increasing global temperatures and the ever-present threat of biological stresses push demand for cultivars with improved stress adaptations. Even with the advent of indoor cultivation, the desire for new cultivars, ideally suited to diverse environments, remains an objective in lettuce breeding. The floral morphology of lettuce promotes cleistogamy, hindering breeding by sexual means, and wild lettuce relatives have a limited gene pool of desirable traits, necessitating trait improvement via biotechnological means [1,2]. CRISPR/Cas9-mediated gene editing in lettuce has been gaining popularity in recent years, with efforts published towards optimization of the technology [3,4,5,6,7]. However, the utilization of biotechnological methods such as CRISPR/Cas9 to improve stress adaptations are dependent upon the ability to efficiently regenerate plants after genetic transformation, thus necessitating the continuous development of ideal transformation and regeneration protocols [8].

Much research has been published in lettuce for the expression of transgenes and downregulation of endogenous genes [1,9,10]. Successful practices have been developed for *Agrobacterium rhizogenes*-mediated hairy root transformation [11,12], transient vacuum infiltration- [13,14,15], electroporation- [16], polyethylene glycol (PEG)- [17], and biolistic bombardment- [18,19] mediated plastid transformation, and stable *A. tumefaciens*-mediated nuclear transformation [2,20,21]. The amenability to tissue culture and transformation, together with a short life cycle, ease of cultivation, and a fully sequenced genome makes lettuce a model vegetable species in molecular biology and biotechnology research, especially in the Asteraceae family [22,23].

Although generic protocols have been developed for a broad range of lettuce cultivars, there remains genotype-dependence for lettuce transformation and regeneration. A broad range of transformation efficiency values (13–97%) have been reported in published protocols [2,24]. Thus, a method ideal for one lettuce genotype may not be optimal for another, and further protocol development is beneficial to the regeneration and transformation of additional lettuce cultivars [2,24,25].

When developing improved transformation or regeneration protocols, it is necessary to test a variety of cultivars [20]. In the present work, a new transformation and regeneration protocol was developed through the study of eleven elite lettuce cultivars, which belong to three of the seven main types of lettuce, i.e., butterhead, crisphead, Latin, leaf, oilseed, romaine, and stem [20]. The ‘Kahu’ and ‘Rosalita’ cultivars belong to the romaine type, characterized by long, upright, and oval dark green leaves [26,27]. The ‘Cocarde’, ‘Green Wave’, ‘Red Sails’, and ‘Royal Oak Leaf’ cultivars belong to the leaf type with a loose rosette [20,28,29,30]. The ‘Bronze Mignonette’, ‘Cobham Green’, ‘Girelle’, ‘Lollo Biondo’, and ‘Mariska’ cultivars are butterhead-type with a loosely formed rosette and soft leaves [26,28,31].

The eleven lettuce cultivars utilized in this study were chosen based upon previous demonstrations of transformation or regeneration success. The ‘Green Wave’ cultivar has been successfully transformed with the *Arabidopsis thaliana HSP17.8* gene [32], the toxin B subunit from *Escherichia coli* towards the development of edible vaccines [29], and a chloroplast-expressed fructose gene from cyanobacteria to enhance the photosynthetic capacity [18]. Both the ‘Cocarde’ and ‘Girelle’ cultivars have been used to express lettuce mosaic virus coat protein [28], and the ‘Cobham Green’ cultivar was utilized in the inaugural transformation of lettuce [21]. The ‘Mariska’ cultivar has been used to express the *Tnt1* retrotransposon from tobacco [31], to express *Green Fluorescent Protein* (*GFP*) and *β-glucuronidase* (*GUS*) reporter genes in transient agroinfiltration assays [15], and to silence the downy mildew resistance gene *Resistance Gene Candidate2* (*RGC2*) by RNAi [33]. In addition, studies have been undertaken to develop tissue culture regeneration methods in the cultivars used in the present study. For example, ‘Red Sails’ and ‘Bronze Mignonette’ were utilized to develop regeneration protocols from mature leaf and cotyledon explants [30,34], and the cotyledons of the ‘Cobham Green’ cultivar were subject to a regeneration study ahead of initial transformation attempts [35]. Regeneration has also been carried out on the cotyledons of ‘Rosalita’, ‘Royal Oak Leaf’, ‘Bronze Mignonette’, ‘Cobham Green’, ‘Lollo Biondo’ [26], ‘Rosalita’, ‘Bronze Mignonette’, and ‘Cobham Green’ [36].

In the present study, different plant growth regulators (PGRs) and selection antibiotics were studied in the aforementioned eleven lettuce cultivars towards the goal of optimizing transformation and regeneration methods. This work demonstrates that an optimized protocol was developed for *Agrobacterium*-mediated transformation and regeneration of these cultivars and paves the way for future works aimed at cultivar development to address biotic and abiotic stress resistance.

## 2. Results

### 2.1. Test of the Effects of PGRs on Callus Induction and Shoot Regeneration

We first sought to identify a media composition, specifically that of the PGRs, that would favor shoot regeneration of the eleven lettuce cultivars in tissue culture. A literature review of shoot regeneration studies across lettuce cultivars indicated that a base callus induction medium containing Murashige and Skoog (MS), 3% sucrose, and 2 g/L Gelzan^TM^ with a pH of 5.8 has been most effective [20] even though the ideal PGR combinations and their success in regeneration have been reported to be highly genotype-dependent (Tables S1 and S2) [10,30,37]. Using this base callus induction medium [20], a PGR trial was first performed on the explants (i.e., non-transformed cotyledons and the first leaf) of 7-day-old seedlings of the romaine-type ‘Kahu’ to identify the ideal PGR combination for callus induction and shoot regeneration. Nine combinations with different auxins (indole-3-acetic acid (IAA) and 1-naphthaleneacetic acid (NAA)) and cytokinins (kinetin and 6-benzyladenine (BA)) were studied (Table 1). The effects of the PGRs were assessed on their ability to develop a callus and the rate at which shooting occurred.

**Figure 1:**
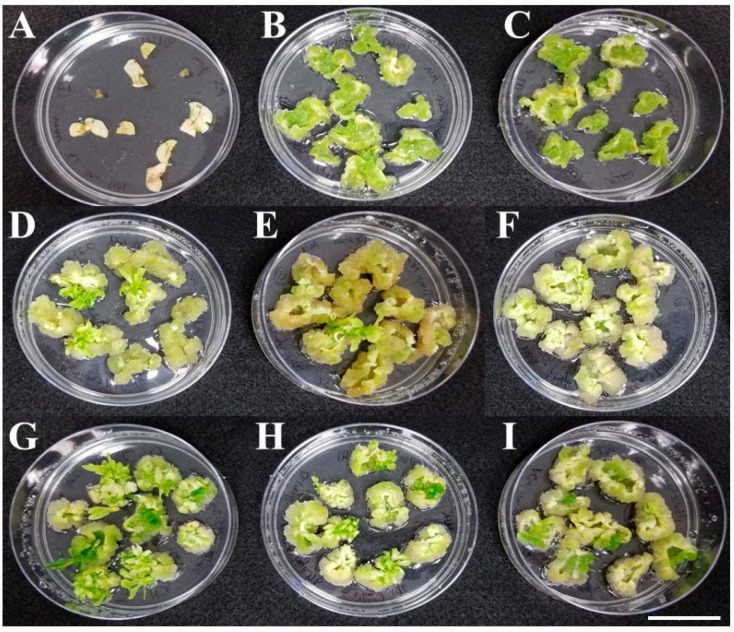
Representative images of the effects of plant growth regulators (PGRs) on callus induction and shoot regeneration from the cotyledon and first leaf explants of 6- or 7-day-old seedlings of non-transgenic lettuce ‘Kahu’ at 28 days after callus induction. Media #1 (**A**), #2 (**B**), #3 (**C**), #4 (**D**), #5 (**E**), #6 (**F**), #7 (**G**), #8 (**H**), and #9 (**I**) with PGR components are listed in Table 1. Each plate is a representative of three replicates for each medium. Bar = 3 cm.

As expected, tissue yellowing and senescence were observed in the control media without PGRs (media #1) with non-transformed ‘Kahu’ explants (Figure 1A). The callus induction media #2 and #3 containing IAA (2.00 and 1.00 mg/L, respectively) and kinetin (1.00 and 0.5 mg/L, respectively) exhibited bright green callus growth but limited shooting (Figure 1B,C). The callus induction media #4–#6 contained the same concentration of NAA (0.05 mg/L) and increasing concentrations of BA (0.25, 0.50, and 1.00 mg/L, respectively). While the media #4 calli appeared mostly green with some yellowing, the media #5 and #6 calli tended towards white and brown, indicating a reduction in health with increased concentration of BA (Figure 1D–F). Callus induction media #7–#9 contained the same concentration of NAA (0.10 mg/L), also with increasing concentrations of BA (0.25, 0.50, and 1.00 mg/L, respectively). Media #7 yielded mostly green and bright yellow calli with a visible shift in preference for shoot induction, while media #8 and #9 yielded white and brown calli with reduced shooting (Figure 1G–I). Thus, media #7 appeared most successful in regenerating shoots and healthy calli.

**Figure 2:**
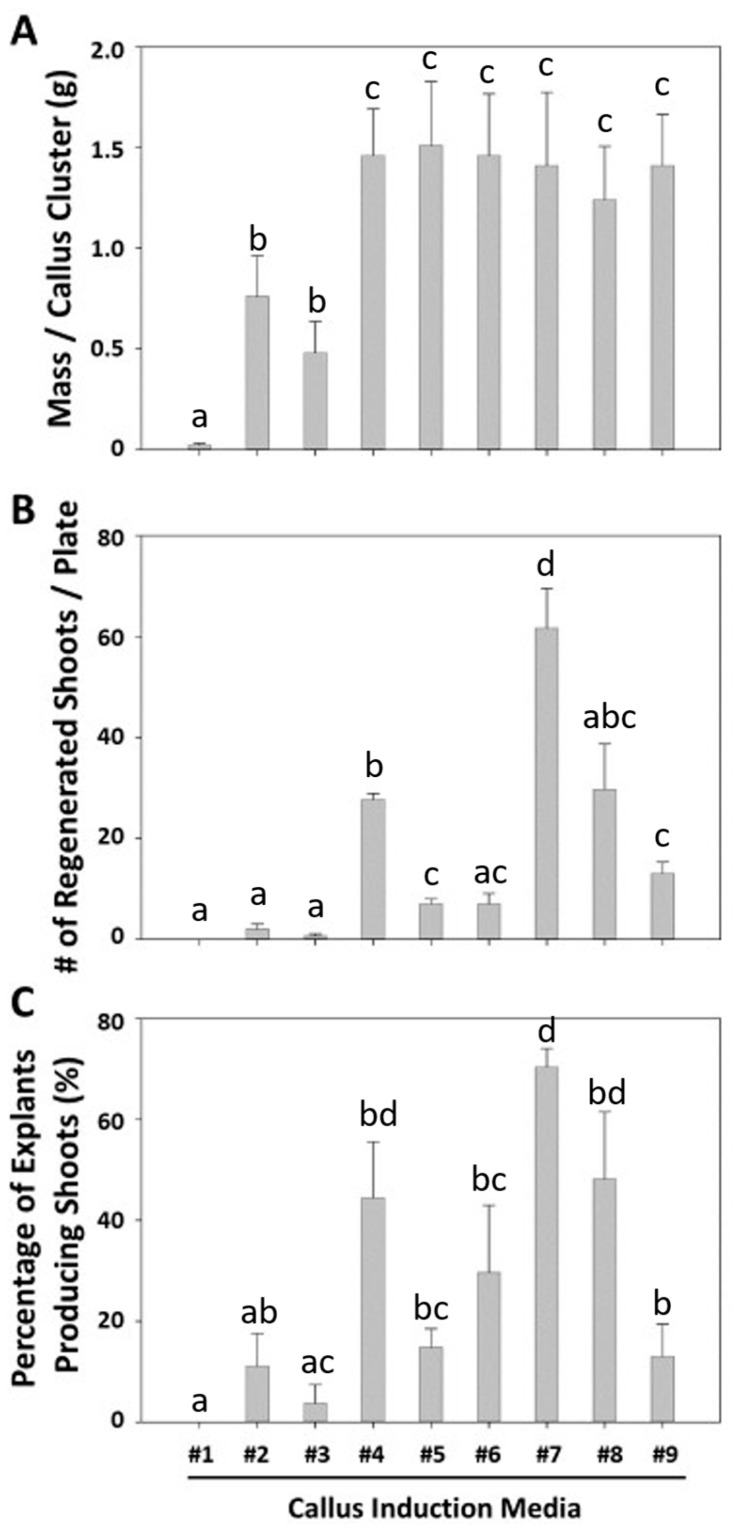
The effects of plant growth regulators on callus induction (**A**), shoot regeneration (**B**), and the percentage of shoot-producing explants (**C**) from the cotyledon and first leaf explants of 6- or 7-day old seedlings of non-transgenic lettuce ‘Kahu’ at 28 days after callus induction. Each value in (**A**) was the average of 9 callus clusters for each treatment. Each value in (**B**) was the average number of regenerated shoots per plate (n = 3) for each treatment. Each value in (**C**) was the average percentage of shoot-developing explants per plate (9 explants/plate for 3 replicate plates) for each treatment. Error bars with compact letter (a-d) displaying groupings represent significant differences based on one-way ANOVA (*p* ≤ 0.05). The *x*-axis indicates the different callus induction media, with treatment #1 serving as the PGR-free control.

We also measured the callus yield, shoot number per plate, and percentage of shoot-producing explants of non-transformed ‘Kahu’ on each medium at 28 days after callus induction. We found that callus induction media #4–#9, containing NAA and BA, yielded a significantly greater mass of calli per callus cluster than media #2 and #3 containing IAA and kinetin (Figure 2A). However, no statistical difference in the callus mass was observed when the concentrations of auxin or cytokinin differed among the NAA/BA media (#4–#9) or between the IAA/kinetin media (#2 and #3) (Figure 2A). When the number of shoots generated per media was scored, media #7 was the best, averaging 61.67 ± 13.58 shoots per plate (Figure 2B). It was also observed that the use of NAA and BA in the media (#5–#9) was more successful at generating shoots than that of the IAA and kinetin media (#2 and #3). In addition, the average number of shoot-producing explants was assessed (Figure 2C). While several of the media resulted in shoot induction, media #7 yielded the greatest percentage of shoot-producing explants (70.37% ± 6.42%). As a result, callus induction media #7 was the best in producing healthy calli and shoots in the romaine-type cultivar ‘Kahu’, and was selected for use in the subsequent transformation work.

### 2.2. Antibiotic Selection in Shoot Induction Media

At the same time, we also tested the effects of the antibiotics hygromycin and kanamycin on the selection of transformed and non-transformed lettuce tissues. As hygromycin has been used for the transformation of ‘Ahvaz’ [50], a romaine-type cultivar [51], hygromycin was chosen for the kill curve analysis in the romaine-type ‘Kahu’ in the present study. We tested the effect of hygromycin with differing concentrations (0, 10, 15, 20, and 25 mg/L) in two differing callus induction media (e.g., media #3 and #7) on non-transformed cotyledons and the first leaf of ‘Kahu’ (Table 2). On media #3, no callus growth occurred at 20 and 25 mg/L hygromycin, while proliferative callus growth was achieved at concentrations of 0 and 10 mg/L, and moderate callus growth was observed at 15 mg/L hygromycin. Similarly, on media #7, bleaching and necrosis in addition to no callus growth were observed at higher hygromycin concentrations (20 and 25 mg/L), while moderate proliferation was observed at 10 mg/L hygromycin, and minimal proliferation was observed at 15 mg/L hygromycin. As a result, 15 mg/L hygromycin was selected for use in subsequent studies in order to avoid potential transgene escape at a lower hygromycin concentration.

As kanamycin has been used for the transformation in the leaf-type ‘Cocarde’ [28] and the butterhead-type ‘Cobham Green’ [21,26], kanamycin was chosen for the kill curve analysis on both transformed and untransformed tissue of ‘Red Sails’ (a leaf type) and ‘Girelle’ (a butterhead type) on callus induction media #3 in the present study. Concentrations of 0, 40, 80, 120, and 200 mg/L of kanamycin were tested, and a visual assessment was performed (Table 2). When no antibiotics were used, a strong proliferation of calli and shooting was observed in both cultivars. Kanamycin concentrations of 120 mg/L and 200 mg/L led to the total necrosis of untransformed tissues, with transformed tissues retaining some greenness but showing no callus growth (Table 2). While minimal callus growth was observed in the transformed tissues of both cultivars at 80 mg/L kanamycin, no growth was observed in the non-transformed tissues. When the non-transformed ‘Red Sails’ was under 40 mg/L kanamycin selection, no growth was observed, while minimal-to-moderate callus growth was observed in the transformed tissues. The ‘Girelle’ cultivar fared similarly, showing minimal to no growth in the non-transformed tissue and moderate proliferation under 40 mg/L kanamycin selection (Table 2). As a result, 40 mg/L kanamycin was the lowest concentration to kill off untransformed tissues and was used in the successive transformation studies.

### 2.3. Agrobacterium-Mediated Stable Transformation of the Tested Lettuce Cultivars

To conduct *Agrobacterium*-mediated stable transformation of the eleven selected cultivars, *Agrobacterium* strains (LBA4404 [2,52] and GV3101 [46,53,54]), co-cultivation time (2 days; [39,52]), explant type (cotyledons and the first leaves; [9,10]), seedling age (6 or 7 days after seeding; [9,10]), and rooting media (PGR-free rooting media; [53]) from published literature were used as reference (Table S1). We first tested the use of 15 mg/L hygromycin for *Agrobacterium*-mediated stable transformation on the 50 explants from 25 seedlings of each of the eleven lettuce cultivars with the GV3101 strain containing the pGFP-GUSPlus plasmid. The *GUSPlus* reporter gene contains an intron, allowing GUS expression only in eukaryotic cells [55,56,57,58]. Utilizing our optimized lettuce transformation method as stated above, a wide array of successful findings were observed (Table 3). We found that hygromycin selection worked successfully for the two romaine-type cultivars ‘Kahu’ and ‘Rosalita’. In ‘Kahu’, a 40.0% regeneration efficiency was achieved with 10 plants regenerated. Half of the regenerated plants were positive for *GUS* expression, as detected by histological GUS staining and PCR confirmation. Thirty-seven plants were regenerated in ‘Rosalita’ with a 152.0% regeneration efficiency, while a mere 24.3% of them were GUS- and PCR-positive.

**Figure 3:**
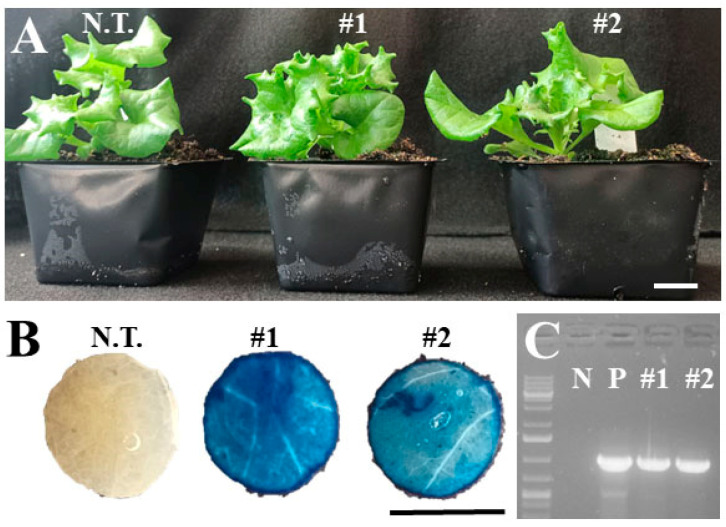
Representative images of constitutive *GUS* expression in transgenic ‘Red Sails’ lines expressing *35S:GUSPlus*. (**A**) Representative plants of non-transformed (i.e., wild-type) and T_1_ GUS-positive line #1 and #2. (**B**) Confirmation of transgene expression using histological GUS staining. (**C**) PCR amplification of the *GUSPlus* gene with primers GusPlus-F and GusPlus-R. N.T., non-transformed. N, negative control (water). P, positive control (plasmid). Bar = 1 cm.

**Figure 4:**
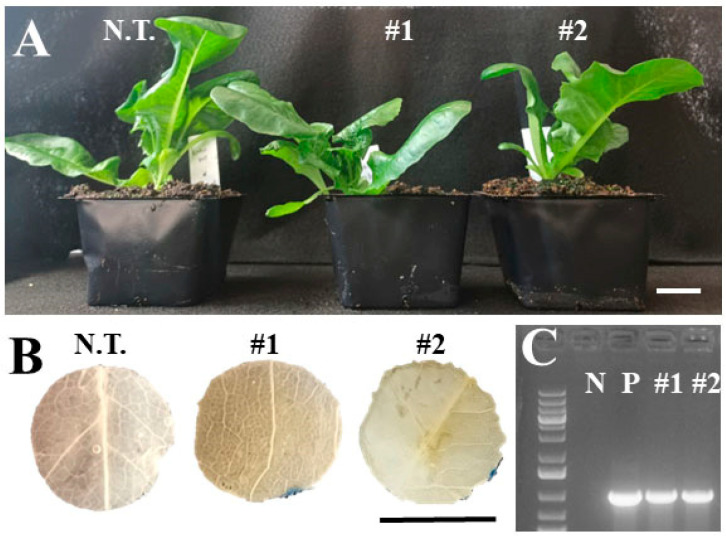
Representative images of reduced *GUS* expression in transgenic ‘Kahu’ lines expressing *35S:GUSPlus*. (**A**) Representative plants of non-transformed (i.e., wild-type) and T_1_ GUS-positive line #1 and #2. (**B**) Confirmation of transgene expression using histological GUS staining. (**C**) PCR amplification of the *GUSPlus* gene with primers GusPlus-F and GusPlus-R. N.T., non-transformed. N, negative control (water). P, positive control (plasmid). Bar = 1 cm.

We also observed that hygromycin selection worked well for three of the four leaf-type cultivars (Table 3). The ‘Red Sails’ cultivar produced 53 regenerants with a 212.0% regeneration efficiency, 69.8% of which were GUS- and PCR-positive. The ‘Green Wave’ and ‘Royal Oak Leaf’ cultivars also delivered a 60.0% and 80.0% regeneration efficiency, respectively, with 100.0% and 50.0% of them positive for GUS and PCR, respectively. However, no plants were regenerated from the ‘Cocarde’ cultivar. Moreover, we noticed an array of intensity in the GUS staining in the transgenic lines, with some transgenic lines accumulating vivid blue coloration across the leaf disc (Figure 3), while other transgenic lines showed spotted GUS staining, e.g., only at the edges of leaf discs (Figure 4), indicating gene silencing from the *35S* promoter, as reported [24,31].

Unfortunately, hygromycin selection showed reduced success in the butterhead-type cultivars (Table 3). Limited regeneration and transformation efficiencies were achieved for the ‘Cobham Green’ and ‘Lollo Biondo’ cultivars. In ‘Cobham Green’, two plants were regenerated with a regeneration efficiency of 8.0%, half of which were GUS- and PCR- positive. In ‘Lollo Biondo’, seven plants were regenerated (28.0% efficiency) with 71.4% of them positive for GUS and PCR. However, plants were not regenerated in the other three cultivars, ‘Bronze Mignonette’, ‘Girelle’, and ‘Mariska’. Thus, the hygromycin selection or regeneration media were not ideal for the butterhead cultivars studied in the present work.

Next, we tested the effects of 40 mg/L kanamycin selection in one butterhead cultivar ‘Mariska’ by transforming it with the pGUSPlus plasmid, with ‘Kahu’ for comparison (Table 3). In ‘Kahu’, 10 plants were regenerated, with a 32.0% regeneration efficiency and 42.9% transformation efficiency, similar to its performance under hygromycin selection. Unlike its performance under hygromycin selection, success was observed in the ability to regenerate transgenic plants from ‘Mariska’ under kanamycin selection, with 15 regenerated plants and a 60.0% regeneration efficiency, 64.3% of them being GUS- and PCR- positive. Thus, kanamycin selection may be preferential for transgenic plant selection in butterhead-type cultivars.

## 3. Discussion

### 3.1. The Effects of Agrobacterium Strain and Concentration and Co-Cultivation Period on Lettuce Transformation

While lettuce is not a natural host to *A. tumefaciens*, transformation attempts have been met with success [21]. As the most favored *Agrobacterium* strains for lettuce transformation have been LBA4404 [2,52] and GV3101 [46,53,54], both strains were utilized in the present study. The successful use of LBA4404 has been achieved in >43 cultivars to date [2,52]. In addition, EHA105 has also been commonly used in lettuce transformation [40,41,43,45,47,59]. Other strains including C58, EHA101, A208, GV3111, and GV2260 have been less commonly used in lettuce transformation [21,28,31,39,60,61,62]. It is worthwhile to test the effects of other *Agrobacterium* strains on the transformation ability in ‘Bronze Mignonette’, ‘Cocarde’, and ‘Girelle’ in future work.

The concentration of *A. tumefaciens* cells is a critical component in the success of lettuce transformation [24]. A study using an array of cultivars observed that a 1:10 dilution of *Agrobacterium* cells from a concentration of OD_600_ 1.1–1.6 yielded more transgenic plants [24]. Thus, the *Agrobacterium* concentration and dilution recommendations in Curtis et al. [24] were followed for all transformations in the present study. In addition, the use of acetosyringone in lettuce transformation has been observed to add no benefit [39], so was not utilized in the present study.

The period of co-cultivation is also critical in enabling lettuce transformation by *A. tumefaciens*. When 1–5 days of co-cultivation were tested in ‘South Bay’, it was reported that transformation was most successful using 3 or fewer days, as longer co-cultivation periods were observed to inhibit later shoot formation [39]. When 2, 4, and 6 days of co-cultivation were tested in ‘Kayser’, 2 days of co-cultivation were found to be the ideal period, while up to 5 days were effective in the leaf explant tissue of ‘Cocarde’ and ‘Girelle’ [28,52]. When 2, 3, and 4 days of co-cultivation were tested in ‘Solan Kriti’, 3 days was found to be most efficient at generating transformants [44]. Due to the enhanced efficiency observed under shorter co-cultivation periods, a 2-day co-cultivation period was utilized in the current study. As our protocol was unsuccessful in regenerating transgenic plants from ‘Cocarde’ and ‘Girelle’, and it was reported that up to 5 days of co-cultivation were effective in the leaf explants of these two cultivars [23], future efforts may find success with longer co-cultivations.

### 3.2. Confounding Effects of PGRs, Antibiotic Selection, and Explant Tissues

In a work studying regeneration alone with 0.1 mg/L IAA, 0.5 mg/L kinetin, and 0.05 mg/L zeatin media, regeneration was successful for ‘Bronze Mignonette’, ‘Lollo Biondo’, and ‘Rosalita’, while ‘Cobham Green’ regenerants did not develop roots and ‘Royal Oak Leaf’ regenerants did not develop shoots [26]. In the present study, regeneration rates differed, with ‘Rosalita’ and ‘Royal Oak Leaf’ successfully regenerating plantlets at a rate of 152% and 80%, respectively, while ‘Lollo Biondo’ (28%), ‘Cobham Green’ (8%), and ‘Bronze Mignonette’ (0%) exhibited reduced success. Similar work towards the development of the ideal PGR combination in shoot regeneration of untransformed tissues arrived at ~0.1 mg/L NAA and ~0.1 mg/L BA as the ideal combination when 15 lettuce cultivars were tested [36]. Among the cultivars tested, ‘Rosalita’, ‘Cobham Green’, and ‘Bronze Mignonette’ generated the most shoots under this PGR combination, with 62–78% explants generating shoots [36]. Altogether, these works demonstrate that a wide degree of success was achievable in different cultivars under differing regeneration media, and the results in the current study, utilizing a differing protocol, attests to that.

Recalcitrance, observed across the lettuce genotypes, has been overcome with protocol modifications. A study to identify the ideal PGR combination to utilize in transformation-recalcitrant lettuce cultivars ‘Red Romaine’ and the butterhead ‘Bibb’ suggested 0.1 mg/L IAA combined with 1 mg/L N-6(2-isopentenyl)-adenine (2ip) in an initial medium, followed by regeneration in media supplemented with 0.05 mg/L NAA and 0.4 mg/L BA [63]. Work to regenerate the recalcitrant leaf-type cultivar ‘Rutgers Scarlet Lettuce’ achieved success when activated charcoal was added to the media, together with 0.5 mg/L NAA and 10 mg/L BA [25]. Similarly, both NAA and BA were identified to be the best combination in the present study, although the optimal concentrations of both PGRs differed. In the present work, we found that the ideal combination of PGRs for shoot regeneration of non-transformed ‘Kahu’ was 0.10 mg/L NAA and 0.25 mg/L BA in media #7. Increased stability in culture has been observed in synthetic PGRs (NAA and BA) over their natural counterparts (IAA and kinetin), possibly contributing to the increased success in generating shoots observed with NAA and BA in the present work, which has been reported previously [64,65]. This PGR combination and concentration have been successfully utilized in work transforming the calf thymus *Thymosin α1* (*Tα1*) gene into the lettuce cultivar ‘Zhouye’ [45] and for the expression of *Oryza sativa chitinase* in the lettuce cultivar ‘Solan Kriti’ [44].

Kanamycin is most commonly used as a selective agent for the transformation of lettuce and has been tested thoroughly in an array of cultivars [2,10], with the effective concentrations of the antibiotics varying from cultivar to cultivar in different studies. The most effective concentrations of kanamycin were 50 mg/L in ‘Cobham Green’ [21], 50 and 100 mg/L in ‘South Bay’ [39], and 100–250 mg/L in ‘Kayser’ [52], although it was reported that 100 mg/L kanamycin was more efficient than 50 mg/L for the genotype-independent transformation of lettuce [2,24]. Hygromycin is a less commonly used antibiotic in the selection of transformed lettuce tissues, but has also been proven to be successful in selecting for positive transformants. For example, the most effective concentrations of hygromycin were 10 mg/L in ‘Solan Kriti’ and ‘Veronica’ [44,66], 15 mg/L in ‘Ahvaz’ [57], and 20 mg/L in ‘Chongchima’ [67]. Together, these works suggest that differing the kanamycin and hygromycin concentrations may be ideal for different cultivars, which was further confirmed in the present study.

The ability to develop quality calli and subsequent shooting differs greatly when tissue is untransformed and under no selection, versus having been transformed and under selection, as was the case in the present study. This observation was made in initial transformation attempts with the ‘Cobham Green’ cultivar as well [21]. While the ideal PGR combinations were developed under no selection pressure, minimal regeneration was observed when kanamycin (50 mg/L) was present [21]. The authors concluded that antibiotics might be slowing the development of transformed tissues and that cultivars showing favorable regeneration ability when not transformed may yet still be recalcitrant to transformation or may decline in regeneration when under selection [21]. Similarly, when differing concentrations of NAA (0.05 and 0.1 mg/L) and BA (0.1, 0.2, and 0.4 mg/L) were tested, the ideal combination for regeneration when paired with 20 mg/L kanamycin was 0.05 mg/L NAA and 0.4 mg/L BA, yielding up to 48% shooting [53]. Even still, the highest transformation efficiency achieved was 19%, when transforming an Iranian landrace with *GTP cyclohydrolase I* (*GCHI*) [53]. Similarly, when the regeneration of transformed leaf tissues was examined with ‘Cocarde’, ‘Girelle’, and ‘Mariska’, the ideal PGR combination was found to be 0.3 mg/L BA and 0.3 mg/L IAA, yielding 50–75% regeneration and a 12–33% transformation efficiency under kanamycin selection (200 mg/L) [28]. While a similar regeneration percentage (60%) was observed in ‘Mariska’ under kanamycin selection in the present study, no plants were regenerated from ‘Cocarde’, ‘Girelle’, or ‘Mariska’ under hygromycin selection. This serves to further indicate the role that selection antibiotics play in confounding tissue regeneration efficiency, and suggests that successive transformation attempts with ‘Cocarde’ and ‘Girelle’ may find success under kanamycin selection, as was observed in ‘Mariska’, with a 64.3% transformation efficiency (Table 3). Antibiotic types and concentrations were not tested across all of the cultivars in the present study. These may have led to both a reduced regeneration efficiency in cultivars more susceptible to antibiotics and reduced the transformation efficiency in cultivars more resistant to antibiotic selection. Future optimization work could optimize the ideal antibiotic type and concentration for the cultivars of choice.

When PGRs were tested in transformed ‘Chongchima’ cotyledons under selection with 20–25 mg/L hygromycin, 0.05 mg/L kinetin, and 0.05 mg/L NAA, a success rate of 10.8% was achieved [67]. Vanjildorj et al. [67] noted the importance of the ratio of cytokinin to auxin (with a ratio of 5:1–10:1 being ideal) rather than the concentration alone. In the present study, the most successful PGR combination for ‘Kahu’ was media #7, which provided a ratio of cytokinin to auxin of 2.5:1 (0.1 mg/L NAA and 0.25 mg/L BA; Table 1). It may be possible that the ideal ratio differs in the other tested cultivars and may approach the suggested 5:1–10:1 ratio [67], and an enhanced regeneration ability can be realized by further protocol development.

Commonly, rooting takes place in PGR-free media [2,37,48]. Interestingly, when differing rooting medium compositions were tested, the medium producing the greatest root regeneration (up to 85%) was an MS base with just sucrose and selective antibiotics [53]. The addition of auxin to the medium reduced the efficiency to between 0 and 10%. Thus, in order to facilitate root development, PGRs were not added to the rooting media in the present study.

We found that one cultivar (‘Red Sails’) proved considerably amenable to the transformation and regeneration methods put forth in the present study, with a 212.0% regeneration rate and a nearly 70% transformation rate when the cotyledon and first leaf were used as the explants (Table 3). Utilizing combinations of IAA, kinetin, and zeatin PGRs, previous regeneration studies with ‘Red Sails’ observed 46% of cotyledon explants developing shoots [30] and 14% of mature leaf explants developing somatic embryos [34]. Thus, the combination of NAA and BA, hygromycin selection, and the cotyledon and first leaf explants worked very efficiently for ‘Red Sails’ in the present study.

While seedlings differing in age have been utilized as explant sources, cotyledons have been the most effective explants for plant regeneration [1,8,20]. A review of lettuce transformation developments cited 2–4 days post-germination as the most productive tissue age for successful callus generation and shooting, and most studies indicated that cotyledons from 3–7-day-old seedlings were the ideal explant for transformation [9,10]. However, there remains a cultivar effect for this preference. A transformation experiment using ‘Cocarde’ and ‘Girelle’ observed that regeneration was slow and minimal when cotyledons were used as an explant, and leaf explants were used instead [28]. As no shoots were regenerated from either cultivar in the present work, this previous study may be one clue as to this failure; the explant tissue and future endeavors towards transformation and regeneration in ‘Cocarde’ and ‘Girelle’ should take this into consideration. While the cotyledons and first leaf from seedlings 6–7 days after planting were utilized as explants for transformation in the present study, leaf explants in these cultivars may be preferable.

Using our optimized protocol, we have successfully achieved a 24.3–100% transformation efficiency for seven of the eleven lettuce cultivars (Table 3). However, our optimized protocol with hygromycin selection was unsuccessful in the four remaining cultivars, i.e., ‘Cocarde’, ‘Girelle’, ‘Bronze Mignonette’, and ‘Mariska’, which had a 0% regeneration and transformation efficiency. The overall poor regeneration ability of ‘Bronze Mignonette’ has been highlighted previously, however [26]. As the replacement of hygromycin selection with kanamycin selection enabled the regeneration of plants from ‘Mariska’ in the present study (Table 3), the transformation of these four cultivars may benefit from additional protocol development in the form of the use of a different selection antibiotic [16,28,31], an increased cytokinin-to-auxin ratio [67], or different explant tissue [28].

### 3.3. The Effects of the 35S Promoter and Agrobacterium Strains on Transgene Expression in Lettuce

The relative ease of transformation and regeneration in lettuce is overshadowed by the misexpression of foreign genes, gene silencing from the *35S* promoter, and lack of transmission into subsequent generations, as transgene expression has been reported to be inconsistent, depending upon cultivar [1,22,68]. The *35S* promoter has been shown to be effective in lettuce [10] and *GUS* is the most commonly used reporter gene for lettuce transformation [2]. However, when *35S:GUS* was used as a reporter in the transformation of ‘Mariska’, blue staining could be observed only at the edges of the leaf punches from the transformed leaves [31]. A study looking at the transformation efficiency with *35S:GUS* in 13 different cultivars also observed an array of intensities in GUS staining [24], indicating that there could be a cultivar effect or gene silencing by the hypermethylation at CpG and Cp(A/T)pG on the *35S* promoter [68]. The two plasmids used in the current study enabled the confirmation of transgene expression in transformed tissues via histological GUS staining, and similar results were observed here as well, as anything from blue spotting to deep blue staining across the leaf discs was observed (Figure 3B and Figure 4B). Future studies of the lines produced in this work can answer whether or not the *GUS* expression is maintained in successive generations.

*Agrobacterium* strain dependence on the *35S:GUS* expression has also been observed in transient infiltration assays when the cultivar ‘Mariska’ was used [15]. While the strain 15955 yielded small blue spots across its leaf tissues, the strain C58C1 yielded strong blue staining throughout its tissue, and the GV3101 strain exhibited small-to-moderate blue spotting of the transformed leaf tissues. In the present study, however, the one cultivar (‘Kahu’) with plants regenerated from transformation with different *Agrobacterium* strains (GV3101 and LBA4404) and different plasmids (pGFP-GUSPlus and pGUSPlus) showed no apparent difference in the *35S:GUS* expression (Figure 4B), although confounding variables preclude a true side-by-side comparison.

## 4. Materials and Methods

### 4.1. Plant Materials and Growth Conditions

The seeds of the lettuce cultivars ‘Kahu’ (PI 251501), ‘Rosalita’ (PI 601592), ‘Cocarde’ (PI 657644), ‘Green Wave’ (PI 566589), ‘Royal Oak Leaf’ (PI 595576), ‘Bronze Mignonette’ (PI 615072), ‘Cobham Green’ (PI 612637), ‘Girelle’ (PI 665186), ‘Lollo Biondo’ (PI 617944), and ‘Mariska’ (PI 665196) were obtained from USDA-ARS-GRIN, while the seeds of the lettuce cultivar ‘Red Sails’ were obtained from Johnny’s Selected Seeds (Albion, ME, USA). Seeds were surface-sterilized by immersion in 10% bleach for 30 min, followed by three rinses with sterile double distilled water [24]. Seeds were germinated in high-top, 9 cm, Petri plates (13 seeds/plate) containing ½ MS media base (Product M519, PhytoTech Labs; Lenexa, KS, USA) [69], supplemented with 1% sucrose and solidified with 2 g/L Gelzan^TM^ (PhytoTech Labs; Lenexa, KS, USA), pH 5.8. Seeds were incubated at 23 °C, under a 16 h photoperiod (18 µmol·m^−2^·s^−1^) for 6–7 days until regeneration and/or transformation procedures took place [10].

### 4.2. Test of PGRs

Based on the published studies in the transformation and regeneration of lettuce, nine different media compositions were tested for their ability to regenerate healthy calli and shoots from untransformed cotyledon and first leaf tissues. On the seventh day after planting, the cotyledons and first leaf of ‘Kahu’ were excised, wounded on the abaxial side across the midrib, and plated (3 replicates, 7–8 explants per plate) onto media #1 ~ #9, all having an MS base, 3% sucrose, and 2 g/L Gelzan^TM^ with a pH 5.8: media #1, no plant growth regulators (negative control); media #2, 2 mg/L IAA and 1 mg/L kinetin; media #3, 1 mg/L IAA and 0.5 mg/L kinetin; media #4, 0.05 mg/L NAA and 0.25 mg/L BA; media #5, 0.05 mg/L NAA and 0.5 mg/L BA; media #6, 0.05 mg/L NAA and 1.0 mg/L BA; media #7, 0.1 mg/L NAA and 0.25 mg/L BA; media #8, 0.1 mg/L NAA and 0.5 mg/L BA; and media #9, 0.1 mg/L NAA and 1 mg/L BA (Table 1). ThE PGRs IAA (Product I885), NAA (Product N600), kinetin (Product K750), and BA (Product B800) were purchased from PhytoTech Labs (Lenexa, KS, USA). Plates were maintained under the same conditions as germination plates and were assessed for callus and shoot growth 28 days after induction.

### 4.3. Plant Antibiotic Selection

A kill curve analysis was performed with hygromycin on 6- or 7-day-old untransformed cotyledons and the first true leaf of ‘Kahu’ with differing concentrations of hygromycin (0, 10, 15, 20, and 25 mg/L; Product H397, PhytoTech Labs; Lenexa, KS, USA) on the callus induction media #3 (1 mg/L IAA and 0.5 mg/L kinetin) and #7 (0.1 mg/L NAA and 0.25 mg/L BA). Each test consisted of 3 replicated plates with 7–8 cotyledons per plate.

A kanamycin kill curve analysis was performed using varied concentrations (0, 40, 80, 120, 160, and 200 mg/L) of kanamycin (Product K378, PhytoTech Labs; Lenexa, KS, USA) on 6- or 7-day-old transformed (with the pGUSPlus plasmid) and untransformed cotyledons and first leaves of ‘Red Sails’ and ‘Girelle’ in callus induction media #3 (1 mg/L IAA and 0.5 mg/L kinetin). Each test consisted of 3 replicated plates with 7–8 cotyledons per plate.

### 4.4. Vector Construction

**Figure 5:**
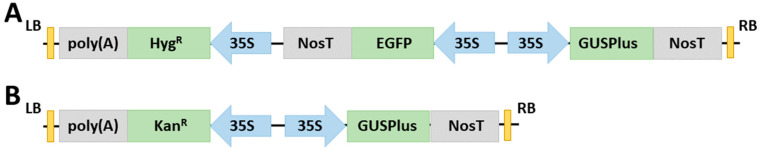
Scheme of the plasmids used in the present study. (**A**) The pGFP-GUSPlus plasmid containing *35S:HygR*, *35S:EGFP*, and *35S:GUSPlus*. (**B**) The pGUSPlus plasmid containing *35S:HygR* and *35S:GUSPlus*. LB, left border; RB, right border.

The pGFP-GUSPlus plasmid (hygromycin selection) was obtained from Addgene (plasmid #64401; hygromycin selection), and contains *GUSPlus* under the control of the *35S* promoter with a *Nos* terminator, *eGFP* under the control of *35S* with a *Nos* terminator, and *HygR* under the control of *35S* with a CaMV pol(A) signal (Figure 5A).

The pGUSPlus plasmid (kanamycin selection) was generated as follows. The *35S:GusPlus-NosT* fragment was extracted from the pZP35SGusPlus plasmid [55] with the help of *EcoR*I and *Hind*III, and inserted into *EcoR*I- and *Hind*III-digested pCAMBIA2200 plasmid. Digestion products were purified from an agarose gel using the QIAquick^®^ Gel Extraction Kit (QIAGEN; San Diego, CA, USA). Ligation products were transformed into *E. coli* DH5α competent cells using the freeze–thaw method [70]. Colony PCR was performed with primers M13F and M13R (Table 4). Positive colonies were increased for plasmid extraction with the QIAprep^®^ Spin Miniprep Kit (QIAGEN; San Diego, CA, USA) for subsequent Sanger sequencing with the M13F primer. The pGUSPlus T-DNA contains *GusPlus* under the control of the *35S* promoter with a *Nos* terminator and *Kan^R^* under the control of *35S* and a CaMV poly(A) signal (Figure 5B).

### 4.5. Agrobacterium-Mediated Lettuce Transformation

The pGFP-GUSPlus and pGUSPlus plasmids were transformed into the GV3101 and LBA4404 strains of *A. tumefaciens*, respectively, using the freeze–thaw method [71]. Transformed *A. tumefaciens* cells were then plated onto solid YEP media supplemented with 100 mg/L rifampicin, and 20 mg/L chloramphenicol or 50 mg/L kanamycin. Colony PCR with M13F and M13R primers was performed on the resulting colonies to check for the presence of the plasmids. A single PCR-positive *Agrobacterium* colony was selected for each plasmid, and increased in 3 mL liquid YEP supplemented with 100 mg/L rifampicin and selection antibiotics at 28 °C overnight. Then, a 200 μL aliquot was transferred to 20 mL YEP with the same antibiotics in an Erlenmeyer flask and grown on a shaker at 150 rpm in the dark at 28 °C for 16–24 h until the optical density at 600 nm (OD_600_) reached 1.1–1.6. The cells were centrifuged (20 min, 5000 g), and, in a sterile hood, the pellet was resuspended in 200 mL liquid MS media supplemented with 30 g/L sucrose to a final ratio of 1:10 [24] for lettuce transformation.

Plant transformation procedures were adapted from Curtis et al. [24]. Cotyledons and the first leaf were excised from 6- or 7-day-old seedlings and wounded on the abaxial side with shallow cuts across the midrib. Wounded tissues were stirred gently in the *Agrobacterium* suspension for 10 min, then blotted dry on sterile Whatman blotting paper and placed abaxial-side-down on co-cultivation media, containing MS, 0.25 mg/L BA, 0.1 mg/L NAA, 30 g/L sucrose, and 2 g/L Gelzan^TM^ with a pH of 5.8 (~8 explants per dish, ~25 mL media per plate). Co-cultivation took place in the dark at 25 °C for two days. After co-cultivation, explants were moved to selective callus induction media containing MS, 30 g/L sucrose, 0.25 mg/L BA, 0.1 mg/L NAA, 400 mg/L timentin, 2 g/L Gelzan^TM^, and 40 mg/L kanamycin or 15 mg/L hygromycin (Table 1 and Table 2) with a pH of 5.8 (~33 mL media/plate) and placed in a growth chamber (23 °C, 16 h photoperiod, 18 µmol·m^−2^·s^−1^). Living tissues were sub-cultured every 14 days onto fresh selection media and observed for shoot formation. The initiation of callus took approximately two weeks, and shoots began forming in two weeks or more. Regenerated shoots were excised and cultured onto rooting media (MS, 30 g/L sucrose, 2 g/L Gelzan^TM^, 400 mg/L timentin, and 7.5 mg/L hygromycin or 20 mg/L kanamycin) in Magenta^®^ GA-7 boxes (~50 mL/box), and maintained as roots developed; this was a process taking a minimum of two weeks. After rooting, plantlets were carefully removed from culture, rinsed free of gel media, and potted into potting soil (Sungro Horticulture Sunshine^®^ Mix #1; Sun Gro Horticulture; Agawam, MA, USA). Plantlets were maintained in a humid environment under plastic domes for one week, for hardening off and subsequent testing.

### 4.6. PCR Confirmation of the Presence of the Transgene

PCR was used to confirm the presence of the transgene in the regenerated T_0_ plants. DNA was extracted from leaf discs using the DNeasy Plant Kit (QIAGEN; San Diego, CA, USA). PCR amplification for genotyping was performed with EmeraldAmp^®^ MAX (Takara; Kusatsu, Shiga, Japan) DNA polymerase and primers GusPlus-F and GusPlus-R spanning a 1 kb region of the *GUSPlus* cassette (Table 4).

### 4.7. Histological GUS Staining

Histological GUS staining was used to confirm the expression of the transgene in the regenerated T_0_ plants using the procedure of Jefferson et al. [72]. Leaf punches (~1 cm) were incubated in X-Gluc stain (1 mM X-gluc, 50 mM NaH_2_PO_4_, pH 7.0) overnight (~16 h) at 37 °C in the dark. Samples were then washed in 70% ethanol, with wash being replaced every few hours to clear greenness and de-stain [72]. Stained discs were then visually assessed for the presence of blue staining and representative photographs were taken (Figure 3B and Figure 4B).

### 4.8. Statistical Analysis

Statistical analysis was carried out using Microsoft Excel^®^ software (version 2501). Analysis of variance (ANOVA) was conducted on PGR study data and compact letter display was used to indicate statistically significant differences at the *p* < 0.05 significance level.

## 5. Conclusions

We have developed an optimized transformation protocol utilizing hygromycin selection for seven of the eleven tested lettuce cultivars of the romaine, leaf, and butterhead cultivar types, and this work marks the first successful genetic transformation of the romaine cultivars ‘Kahu’ and ‘Rosalita’, the leaf lettuces ‘Red Sails’ and ‘Royal Oak’ Leaf’, and the butterhead ‘Lollo Biondo’. While this newly developed protocol had limited success in the remaining four cultivars, the utilization of kanamycin selection led to successful transformation and regeneration in one additional cultivar, ‘Mariska’. Further protocol optimization is needed to effectively transform the remaining cultivars and future work would benefit from expanding into other cultivar types or wild relatives of lettuce to increase genetic diversity in lettuce transformation.

## Figures and Tables

**Table 1:** Plant growth regulator (PGR) trial for callus induction and shoot regeneration from the cotyledon and first leaf of the lettuce cultivar ‘Kahu’. Dot (-), not tested.

Media	Present Study			Published Studies		
	Auxin (mg/L)	Cytokinin (mg/L)		Auxin (mg/L)	Cytokinin (mg/L)	Refs.
#1	0.00	0.00		0.00	0.00	
	**IAA (mg/L)**	**Kinetin (mg/L)**		**IAA (mg/L)**	**Kinetin (mg/L)**	
#2	2.00	1.00		5.60	0.68	[38]
#3	1.00	0.50		-	-	[21]
	**NAA (mg/L)**	**BA (mg/L)**		**NAA (mg/L)**	**BA (mg/L)**	
#4	0.05	0.25		0.05	0.20	[39,40,41,42,43]
#5	0.05	0.50		-	-	[35]
#6	0.05	1.00		-	-	[25]
#7	0.10	0.25		-	-	[44,45]
#8	0.10	0.50		-	-	[46,47,48,49]
#9	0.10	1.00		0.10	2.00	[25]

**Table 2:** Visual scoring of callus induction of three lettuce cultivars on hygromycin and kanamycin media. +, minimal callus growth; ++, moderate callus growth; +++, strong callus growth; -, no callus growth; --, no callus growth and necrosis; N.T., non-transformed; and T., transformed.

Media #	Hygromycin (mg/L)					Cultivar	Cultivar Type
	0	10	15	20	25		
**#3**	+++	++	+	-	--	Kahu (N.T.)	Romaine
#7	++	++	+	--	--	Kahu (N.T.)	Romaine
**Media** **#**	**Kanamycin (mg/L)**					**Cultivar**	**Cultivar Type**
	**0**	**40**	**80**	**120**	**200**		
#3	+++	-	-	--	--	Red Sails (N.T.)	Leaf
#3	+++	+	+	-	-	Red Sails (T.)	Leaf
#3	++	-	-	--	--	Girelle (N.T.)	Butterhead
#3	++	++	+	-	-	Girelle (T.)	Butterhead

**Table 3:** Plant regeneration efficiency and transformation efficiency of the eleven lettuce cultivars after *Agrobacterium*-mediated transformation. Regeneration efficiency (%) was calculated by dividing the number of regenerated lines by the number of initial seedlings used for transformation. Transformation efficiency (%) was calculated by dividing the number of GUS- and PCR-positive lines by the number of regenerated lines. Each treatment utilized 50 cotyledon/first leaf explants from 25 seedlings (6 or 7 days old). For transgenic plant selection, pGFP-GUSPlus transformation utilized 15 mg/L hygromycin, and pGUSPlus transformation utilized 40 mg/L kanamycin.

Cultivar	Plasmid	Regenerated Plants /Initial Seedlings	Regeneration Efficiency (%)	Transformation Efficiency (%)
Kahu	pGFP-GUSPlus	10/25	40.0	50.0
Rosalita	pGFP-GUSPlus	38/25	152.0	24.3
Red Sails	pGFP-GUSPlus	53/25	212.0	69.8
Green Wave	pGFP-GUSPlus	15/25	60.0	100.0
Royal Oak Leaf	pGFP-GUSPlus	20/25	80.0	50.0
Cocarde	pGFP-GUSPlus	0/25	0.0	0.0
Girelle	pGFP-GUSPlus	0/25	0.0	0.0
Cobham Green	pGFP-GUSPlus	2/25	8.0	50.0
Lollo Biondo	pGFP-GUSPlus	7/25	28.0	71.4
Bronze Mignonette	pGFP-GUSPlus	0/25	0.0	0.0
Mariska	pGFP-GUSPlus	0/25	0.0	0.0
Kahu	pGUSPlus	8/25	32.0	42.9
Mariska	pGUSPlus	15/25	60.0	64.3

**Table 4:** Primers used in the present study.

Primer Name	Primer Sequence (5′ > 3′)
M13F	TGTAAAACGACGGCCAGT
M13R	CAGGAAACAGCTATGAC
GusPlus-F	GACTGACCATCGATGTCTATG
GusPlus-R	GCCGAAATCTGGAATGTTGGT

## Data Availability

Data are contained within the article and supplementary materials.

## Supplementary Materials

The following supporting information can be downloaded at https://www.mdpi.com/article/10.3390/plants14040620/s1: Table S1: Literature review of *A. tumefaciens*-mediated lettuce transformation methods [73,74,75,76]; Table S2: Literature review of *A. tumefaciens*-mediated lettuce regeneration methods [77].
