# Supplementary material for: An Improved Method for Agrobacterium-Mediated Genetic Transformation of Three Types of Lettuce
Source: Plants (Basel). 2025 Feb 18;14(4):620. doi: 10.3390/plants14040620 (PMC11858900; doi:10.3390/plants14040620)
Supplement: Supplementary file 1 [file plants-14-00620-s001.zip › plants-3441769-supplementary.pdf]

Table S1. Literature review of *A. tumefaciens*-mediated lettuce transformation and regeneration methods

Cultivar	Explant	Explant Age (days)	Antibiotic Conc. (mg/L)	*Agro* Strain	OD600	Co-cultivation Time (days)	Auxin (mg/L)	Cytokinin (mg/L)	Transform. Efficiency (%)	Refs
'Achát'	Cotyledon	10	100 Kan	LBA4404	0.9	1	0.93 NAA	0.56 BA	N.A	[73]
'Zhouye'	Cotyledon	N/A	50 Kan	EHA105	0.5 - 0.6	1.5	0.1 NAA	0.25 BA	N.A	[45]
'Lake Nyah', 'Mantillia', 'Bastion', 'Carvello', 'Cortina', 'Evola', 'Flora', 'Luxor', 'Omega', 'Reflex', 'Danilla', 'Impulse', 'Lobjoits'	Cotyledon	7	50-**100** Kan	LBA4404	1.1 - 1.6	2	0.04 NAA	0.5 BA	N.A	[24]
'Lake Nyah'	Cotyledon	7	100 Kan	LBA4404	N/A	2	0.04 NAA	0.5 BA	N.A	[74]
'Saladin'	Cotyledon	7	50, 100 Kan	LBA4404	1.1 - 1.6	2	0.04 NAA	0.5 BA	N.A	[75]
'Veronica'	Cotyledon	2	10 Hyg	EHA105	N/A	0.01	0.1 IBA	0.1 BA	42.50%	[66]
'Cocarde', 'Girelle', 'Jessy'	Leaf	7	200 Kan	GV2260	N/A	1 - 5	0.3 IAA	0.3 BA	N.A	[28]
'Kayser'	Cotyledon	5	0, **50, 100, 250**, 500 Kan	LBA4404	N/A	**2**, 4, 6	0.05 IAA	0.01 BA	70.00%	[52]
'Iranian landrace'	Cotyledon	3	0, 10, **20**, 30, 40, 50 Kan	GV3101	0.8 - 1.0	1, 2, **4**, 6	**0.05**, 0.1 NAA	0.1, 0.2, **0.4** BA	19.00%	[53]
'Batavia Blonde'	Cotyledon	6	100 Kan	EHA105	0.5 - 0.8	5 - 7	0.1 NAA	0.1 BA	N.A	[59]
'Verônica'	Cotyledon	2	4 PPT	EHA105	0.5 - 1.0	2	0.05 NAA	0.2 BA	N.A	[40]

Table S1. (Cont.)

Cultivar	Explant	Explant Age (days)	Antibiotic Conc. (mg/L)	*Agro* Strain	OD^{600}	Co-cultivation Time (days)	Auxin (mg/L)	Cytokinin (mg/L)	Transform. Efficiency (%)	Refs
'LE126', 'Seagreen'	Cotyledon	6	50 Kan	LBA4404	1.1 - 1.6	2	5.6 IAA	0.68 Kin	85.70%	[38]
N/R	Cotyledon	5 – 7	0.5 PPT	LBA4404	N/R	2	0.1 NAA	0.5 BA	N.A	[48]
'Green Wave'	Cotyledon	5	25 PPT	N/R	N/A	2	0.05 NAA; 2 2,4-D	0.25 Kin	N.A	[32]
'Simpson', 'Red Romaine', 'Bibb'	Leaf	42	100 Kan	LBA4404	1.0	3	**0.05**, 0.1 NAA; 0.1 IAA	1 2ip; 0.4 BA; 1 Zeatin	N.A	[63]
N/A	Cotyledon	7	1 PPT	GV3101	N/A	N/A	0.1 NAA	0.5 BA	N.A	[46]
'Grand Rapids'	Cotyledon	7	100 Kan	LBA4404	N/A	3	0.1 NAA	0.5 BA	N.A	[49]
'Potosina', 'Green Wave'	Cotyledon	7	100 Kan	LBA4404	2.0	2	0.02 NAA	0.5 BA	N.A	[29]
'Mariska', 'Jessy'	Leaf	10	200 Kan	GV2260	N/A	2	0.3 IAA	0.15 BA	N.A	[31]
'Cobham Green'	Cotyledon	4	0, 10, 25, **50**, 100, 250, 500 Kan	GV3111	0.5	2	1 IAA	0.5 Kin	N.A	[21]
'Ahvaz'	Cotyledon	7	0 - 20 (**15**) Kan	LBA4404	0.8 - 1.0	0.08	0.1 NAA	0.1 BA	N.A	[50]
'Grand Rapids'	Cotyledon	1 - 2	300 Kan	LBA4404	5.2	3	0.05 NAA	0.2 BA	N.A	[42]

Table S1. (Cont.)

Cultivar	Explant	Explant Age (days)	Antibiotic Conc. (mg/L)	*Agro* Strain	OD^{600}	Co-cultivation Time (days)	Auxin (mg/L)	Cytokinin (mg/L)	Transform. Efficiency (%)	Refs
'Syrena'	Cotyledon	2 - 3	2.5 PPT	EHA105	1.0	2	0.05 NAA	0.2 BA	N.A	[41]
'Longifolia'	Cotyledon	4-7	40 Kan	EHA105	0.8 - 1.0	1.5 - 2	0.1 NAA	0.5 BA	N.A	[47]
'Solan Kriti'	**Leaf,** petiole	15 - 20	10 Hyg, PPT	LBA4404	0.5	2, **3**, 4	**0.1** NAA	0.75 Kin; **0.25** BA	N.A	[44]
'South Bay'	Cotyledon	**1, 2, 3**, 4, 5	0, 25, **50, 100**, 200 Kan	A208	0.7	**1, 2, 3**, 4, 5	0.05 NAA	0.2 BA	N.A	[39]
'TN-96-39'	Cotyledon	4	10 Kan	LBA4404	0.6	1	0.05 NAA	0.2 BA	N.A	[43]
'Chongchima'	Cotyledon	7	5, 10, **20**, 30, 40, 50 Hyg	EHA105	0.6 - 0.8	3	0.0, **0.05**, 0.1, 0.2, 0.4, 1.6 NAA	0.0, 0.25, **0.5**, 1.0, 2.0 Kin	N.A	[67]
'Crystal'	Cotyledon	7	50 Kan	LBA4404	0.5	2	0.04 NAA	0.5 BA	N.A	[76]

Bold, the best explant age or concentration. Hyg, hygromycin; Kan, kanamycin; Kin, Kinetin; PPT, phosphinothricin; 2,4-D, 2,4-Dichlorophenoxyacetic acid; IBA, indole-3-butyric acid; 2ip, 6-(γ,γ-Dimethylallylamino)purine. Transform. Efficiency, Transformation Efficiency. N.A., not available.

Table S2. Literature review of lettuce regeneration methods

Cultivars tested	Explant	Explant Age (days)	Auxin (mg/L)	Cytokinin (mg/L)	Transform. Efficiency	Refs
‘Bronze Mignonette’, ‘Bambino’, ‘Iceberg’, ‘Cobham Green’, ‘Sweet Butter’, ‘Simpson Elite’, ‘Rosalita’, ‘Paris White’, ‘Lollo Biondo’, ‘Royal Oak Leaf’, ‘Red Salad Bowl’, ‘South Bay’, ‘Black Seeded Simpson’, ‘Lollo Rosso’, ‘Mainspring’, ‘Oak Leaf’, ‘Tango’, ‘Prize Head’, ‘Sangria’, ‘Mini-Green’, 'Nevada', ‘New York Head’	Cotyledon	2	0.1 IAA	0.5 Kin; 0.05 zeatin	N.A	[26]
‘Rutgers Scarlet’, ‘Winter Destiny’	Cotyledon	3	0.05, 0.1, 0.125, 0.15, 0.2, 0.25, 0.3, 0.35, 0.4, 0.45, **0.5 NAA**	0.009, 1.0, 2.0, 3.0, 4.0, 5.0, 6.0, 7.0, 8.0, 9.0 BA; 2.0 Kin; 2.0 thidiazuron; 2.0 zeatin; 2.0, 2.5, 5.0, **10.0 BA**; **200 activated charcoal**	N.A	[25]
‘Cobham Green’, ‘Hilde’, ‘Avondefiance’, ‘Continuity’, ‘Sabine’, ‘Capitan’, ‘Reskia’, ‘Dandie’, ‘Little Gem’, ‘Lobjoits Cos’, ‘Pennlake’, ‘Salad Bowl’, ‘Red Salad Bowl’	Cotyledon and first leaf	7, 14	0.05 NAA	0.5 BA	N.A	[35]
‘Romaine’	Cotyledon, leaf	7, 40	0.03, 0.05, **0.1**, 0.15, 0.17 NAA	0.1, **0.4**, 0.7, 0.82 BA	N.A	[37]

Table 2. (cont.)

**Cultivars tested**	**Explant**	**Explant Age (days)**	**Auxin (mg/L)**	**Cytokinin (mg/L)**	**Transform. Efficiency**	**Refs**
‘Red Oak Leaf’, ‘Red Coral’, ‘Grand Rapids’, ‘Black Seeded Simpson’, ‘Great Lakes’, ‘Greenway’, ‘Bambino’, ‘Webb Wonderful’, ‘Iceberg’, ‘Cos’, ‘Paris White’, ‘Rosalita’, ‘Buttercrunch’, ‘Bronze Mignonette’, ‘Cobham Green’	Cotyledon	3 – 14 (**3-5**)	0 - 1.0 NAA (**0.1** NAA); 0.1, 5.0 IAA	0 - 1.0 BA; 6.88 Kin; 0.05 Zeatin; 0.5 Kin; **0.1 BA**	N.A	[36]
‘Yazd’, ‘Ahvaz’	Cotyledon	**3**, 7	0.02, 0.05, **0.1**, 0.5, 1.0 **NAA**	**0.1**. 0.4, 0.5, 1.0 **BA**	N.A	[77]
All Year Round', 'Great Lakes', 'Greenfields', 'Green Velvet', 'Spring Salad', 'Summer Gem', ‘Webb's Wonderful’, ‘Winter Triumph’, ‘Yatesdale’, ‘Bronze Mignonette’, ‘Buttercrunch’, ‘Green Mignonette’, ‘Sweet Butter’, ‘Tom Thumb’, ‘Red Oak Leaf’, ‘Red Regency’, ‘Red Sails’, ‘Salad Bowl’, ‘Cos’, ‘Green Cos’, ‘White Cos Signal’	cotyledon	5	**0.1 IAA**	**0.5 Kin**; 0.5 Kin; **0.05 zeatin**	N.A	[30]

Bold, the best explant age or concentration. Kin, Kinetin. Transform. Efficiency, Transformation Efficiency. N.A., not available.
